# New insights into *Canis familiaris* papillomaviruses genetics and biology: Is the genetic characterization of CPV types and their variants an important clinical issue?

**DOI:** 10.1590/1678-4685-GMB-2021-0388

**Published:** 2022-09-12

**Authors:** Jordana Dantas Rodrigues Reis, Marcus Vinicius de Aragão Batista

**Affiliations:** 1 Universidade Federal de Sergipe, Centro de Ciências Biológicas e da Saúde, Departamento de Biologia, Laboratório de Genética Molecular e Biotecnologia (GMBio), São Cristóvão, SE, Brazil.

**Keywords:** Canine papillomavirus, dogs, genetic diversity, SCC, variant

## Abstract

*Canis familiaris* papillomavirus (CPV) is a member of the *Papillomaviridae* family and is found in dogs. After infection, the host can remain asymtomatic or develop benign ephitelial neoplasms such as papillomas and pigmented viral plaques, which can progress to cancer, in the form of squamous cell carcinoma (SCC). In humans, 227 types of human papillomavirus (HPV) have been described, with a well-established risk classification for cancer development. In addition, it is also known that variants of some high-risk HPV types may present different risks in respect of SCC development. In dogs, however, only a few types of CPV have been identified, despite the growing interest in this area, and knowledge on the genetic characterization of CPV variants is still scarce. Recent studies of CPV have shown that, as with HPV, benign neoplasia can develop into cancer, but it is believed that there are many more types and variants still to be described. Therefore, the aim of this study was to describe the genetics and biology of CPV, with the focus on what is known about lesions, geographic localization, virus types and variants.

## Introduction

*Canis familiaris* papillomavirus (CPV) belongs to the extensive family *Papillomaviridae* that infect vertebrate hosts such as mammals, birds, reptiles and fish (Van Doorslaer et al., 2018). This family has great genetic diversity but, unsurprisingly, the most frequently studied papillomavirus (PV) has been the human papillomavirus (HPV) (Rector and Van Ranst, 2013; Van Doorslaer, 2013). 

CPV infection is considered species-specific to dogs, but oral papillomatosis has been described in two members of the same *Canidae* family that the subspecies *Canis lupus familiaris* belongs to, namely the wolf and the coyote (Lange and Favrot, 2011). In the USA, a gray wolf with oral papillomatosis was found to be infected with a CPV1 of the species *Lambdapapillomavirus 2* (Knowles et al., 2017). 

Viral transmission occurs by cutaneous or mucosal contact facilitated by some trauma affecting the basal layer of the skin and the mucosal epithelium that results in neoplastic lesions or asymptomatic infection (Lange et al., 2011; Sykes and Luff, 2014; Sardon et al., 2015). The neoplasms that have been associated with CPV are benign exophytic and endophytic papillomas, and pigmented viral plaques, with progression to malign neoplasia in the form of squamous cell carcinoma (SCC) (Zhou et al., 2014). The disease canine papillomatosis mostly affects young, immunosuppressed dogs with some factors influencing the gravity of the clinical status of the animal, such as its genetic background and the pathogenicity of the PV involved (Lange and Favrot, 2011). 

## Genome and infection cycle

**Figure 1 -:**
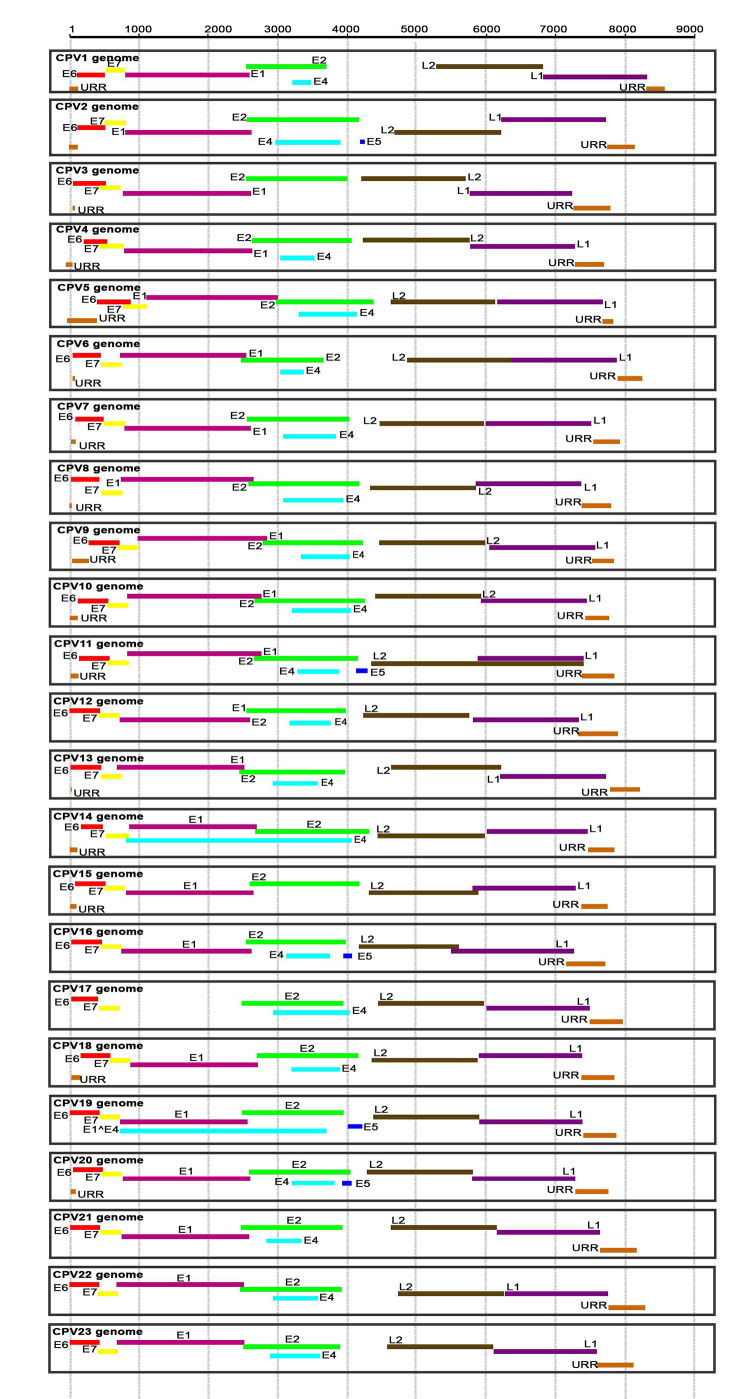
Comparative representation of CPV genomes. The presence, location and length of each gene are represented. The L2 gene sequence of CPV11, available in GenBank, is mutant, overlapping with the L1 gene sequence.

*Papillomavirus (PVs)* are double-stranded DNA viruses with a circular genome that can have up to 8,607 bp with a non-enveloped, 55 nm capsid of icosahedral symmetry (Van Doorlslaer *et al*., 2018). The genome of CPV varies from 7,742 bp (CPV4) to 8,607 bp (CPV1) (Table 1). It has six to eight open reading frames (ORFs): E1, E2, E4, E5, E6, E7 (the early genes), L1 and L2 (the late and structural ones) (Figure 1) (Bernard et al., 2010). Although there are some variations in CPV ORFs, they are translated into proteins that compose the viral capsid and regulate the infection cycle of the virus, which seems to be very similar among all types of *PVs.*

**Table 1 -:** Genomic characterization and length variation for each CPV genotype.

CPV type	GenBank ID	Length	E1	E2	E4	E5	E6	E7	L1	L2
CPV1	GI:9627734	8607	816-2609	2551-3708	3104-3463	______	102-536	533-826	6837-8348	5288-6829
CPV2	GI:56693036	8101	795-2618	2557-4152	2912-3907	4169-4294	105-512	515-811	6202-7713	4662-6188
CPV3	GI:113200740	7801	732-2618	2560-4011	______	______	25-480	440-742	5757-7259	4219-5736
CPV4	GI:164429763	7742	813-2702	2644-4107	______	______	109-564	524-823	5795-7294	4249-5775
CPV5	GI: 255683764	7810	1132-3021	2963-4432	3429-4193	______	422-880	840-1142	6167-7672	4628-6145
CPV6	GI:258611059	8242	706-2517	2459-3625	3036-3389	______	13-426	423-716	6368-7876	4823-6358
CPV7	GI:255683756	7955	774-2591	2530-4044	3068-3802	______	76-492	494-790	6032-7546	4481-6013
CPV8	GI:347750421	7784	742-2619	2561-4144	3084-3908	______	1-435	438-752	5849-7342	4308-5837
CPV9	GI:363540888	7873	970-2835	2777-4240	3348-4001	______	263-718	678-980	6051-7556	4481-5998
CPV10	GI:363540896	7774	844-2724	2666-4258	3189-4022	______	95-532	543-854	5912-7420	4390-5901
CPV11	GI:348659024	7828	826-2715	2657-4141	3228-3902	4131-4316	95-574	534-836	5889-7388	4338-7388
CPV12	GI:388542469	7890	708-2600	2542-4002	3113-3763	______	1-456	416-718	5811-7316	4238-5758
CPV13	GI:402282430	8228	712-2526	2468-3973	2931-3731	______	13-438	435-725	6249-7763	4645-6228
CPV14	GI:430025787	7826	829-2703	2654-4273	829-4037	______	112-522	525-839	5978-7474	4437-5966
CPV15	GI:429841972	7776	783-2663	2605-4170	________	______	1-507	482-793	5864-7357	4278-5852
CPV16	GI:765702648	7796	702-2594	2536-3957	3107-3718	3978-4124	1-456	416-712	5565-7202	4148-5653
CPV17	GI:974142334	8007	________	2462-3952	2928-3707	______	1-411	414-710	6003-7523	4451-5986
CPV18	GI:1046841328	7810	861-2750	2692-4143	3158-3904	______	154-609	569-871	5896-7398	4355-5872
CPV19	GI:1064859043	7941	699-2543	2482-3954	699-3712	4001-4135	1-417	419-715	5934-7445	4382-5917
CPV20	GI:1008264056	7839	762-2660	2602-4068	3173-3829	3907-4068	1-507	467-772	5832-7331	4294-5808
CPV21	GI:1464250060	8225	684-2525	2467-3921	2834-3361	_______	1-429	416-697	6185-7705	4636-6171
CPV22	GI:1464250068	8300	694-2511	2453-3934	2919-3692	_______	1-420	417-707	6321-7835	4737-6305
CPV23	GI:1464250076	8140	694-2517	2459-3931	2922-3689	_______	1-420	417-707	6000-7673	4580-6154

The infection cycle of CPV follows the differentiation cycle of keratinocytes in the epidermis. Initially, the early genes are expressed in the cell nucleus at the basal layer in which the replication of viral DNA occurs. Then, the expression of early genes reduces and the genome becomes episomal, and there is an increase in the expression of genes that leads to cell cycle control (Yhee et al., 2010; Lange and Favrot, 2011; Lange *et al*., 2013). The late genes are expressed in the nucleus of the keratinocytes at the stratum spinosum, stratum granulosum and stratum corneum at the end of cell differentiation. The late genes encode the proteins that are responsible for the production of the viral particles and the assembly of the virions into the nucleus. The viral particles are then released (Lange and Favrot, 2011; Lange *et al*., 2013). 

The E1 and E2 genes, which were present in the ancestor of the papillomavirus, act in viral DNA replication in the nucleus of the host cell. The E2 protein is the most regulatory protein in this cycle, facilitating the binding of E1 to the upstream regulatory region (URR or LCR) between L1 and E6 to begin replication (Van Doorslaer et al., 2018). 

The genome of all *PVs* contains the L1 and L2 genes because they are structural and conserved genes, but some early genes are not present in different viral types (Table 1). The observation of specific early genes presence is important because some of these produce oncoproteins such as E5, E6, and E7. Cases of cancer in BPV1 are associated with E5, and in HPV with E6 and E7 (Yuan et al., 2007; Vande Pol and Klingelhutz, 2013). CPV2 and CPV16 have been isolated in cases of cutaneous SCC in dogs, and they produce the oncoproteins E5, E6 and E7. Although all CPV types have the E6 and E7 oncoproteins, more studies should be done to elucidate the role of each CPV type in respect of cancer development risk, and the role of these oncoproteins in the progression of pre-neoplastic lesions to a malignant neoplasia. In this context, to the best of our knowledge, only CPV1, CPV2, CPV3, CPV7, CPV9, CPV12, CPV15, CPV16, and CPV17 have been isolated in cases of cancer (Lange et al., 2016; Thaiwong et al., 2018; Chang et al., 2020b). 

The E2 protein may influence the development of cancer in lesions caused by CPV9. Analysis of CPV9 genomes isolated from benign and malignant SCC lesions showed that the nucleotide sequence of the virus from the malignant lesion presents a 328 bp deletion at the 3’end in the gene E2. In cases of cancer due to HPV, E2 deletion results in an increase in E6 and E7 protein expression; however, in a case of SCC due to CPV9 no change in the mRNA expression of E6 and E7 was found, indicating that other mechanisms were responsible, for example, differences in protein translation or stability (Chang et al., 2020b).

## Classification and taxonomy

The L1 gene encodes the major viral capsid protein, the L1 protein, which is the main component of the viral particle used for vaccine production. Furthermore, the L1 gene is used for papillomavirus classification and construction of phylogenetic trees (Bernard et al., 2010). Originally, the classification of *PVs* was based on the similarity between L1 nucleotide sequences. The result of the genetic distance observed from an alignment of multiple sequences and the construction of phylogenetic trees was used to classify the *PVs* into genus, species, types, subtypes and variants. Different genera share less than 60% identity; different species in the same genus share between 60% and 70% identity; and the identification of a new type occurs when the differences between the nucleotide sequences are greater than 10% compared to the closest known *PV* type (De Villiers et al., 2004; Van Doorslaer et al., 2017). 

In addition to the classification method suggested by De Villiers et al. (2004), which is still accepted, the most recent taxonomy report about the *Papillomaviridae* family from The International Committee on Taxonomy of Viruses (ICTV) takes into account the visual inspection of phylogenetic trees based on L1, L2, E1 and E2 genes as a genus distinction criterion (Van Doorslaer et al., 2018). 

The ICTV is responsible for *Papillomaviridae* family nomenclature, classifying them into subfamilies, genera and species. The classification criteria for subfamilies also consider the identity of L1 nucleotide sequences, where different members of the subfamilies share less than 45% identity in the L1 gene (Van Doorslaer et al., 2018). 

The scientific community adopted the classification of *PVs* into types, subtypes and variants. The classification of *PVs* into types is based on the similarity between L1 gene sequences. However, to classify a variant (encompassing the subtype), the complete genome sequences must be analyzed: differences of less than 10% define a new variant; differences of 1% or more between variants of the same type defines the lineages, and differences of 0.5 to 1% define the sublineages (Burk et al., 2013; Chen et al., 2015).

In addition, the nomenclature approved by the ICTV distinguishes two subfamilies: *Firstpapillomavirinae* and *Secondpapillomavirinae*. All CPV types belongs to the subfamily *Firstpapillomavirinae*, and are named based on the Greek alphabet. The species have the same name as the genus plus an Arabic number after the name to differentiate it. The CPV genera and species approved by the ICTV are shown in Table 2.

**Table 2 -:** CPV genus, species, and types. Species approved by ICTV.

Name	Abbreviation	Genus	Species	References
*Canis familiaris oral Papillomavirus 1*	CPV1	*LambdaPV*	*LambdaPV 2*	Munday et al., 2016; Rector and Van Ranst, 2013
*Canis familiaris Papillomavirus 2*	CPV2	*TauPV*	*TauPV 1*	Munday et al., 2016; Rector and Van Ranst, 2013
*Canis familiaris Papillomavirus 3*	CPV3	*ChiPV*	*ChiPV 1*	Munday et al., 2016; Rector and Van Ranst, 2013
*Canis familiaris Papillomavirus 4*	CPV4	*ChiPV*	*ChiPV 2*	Rector and Van Ranst, 2013
*Canis familiaris Papillomavirus 5*	CPV5	*ChiPV*	*ChiPV 1*	Rector and Van Ranst, 2013
*Canis familiaris Papillomavirus 6*	CPV6	*LambdaPV*	*LambdaPV 3*	Rector and Van Ranst, 2013
*Canis familiaris Papillomavirus 7*	CPV7	*TauPV*	*TauPV 1*	Munday et al., 2016; Rector and Van Ranst, 2013
*Canis familiaris Papillomavirus 8*	CPV8	*ChiPV*	*ChiPV 3*	Rector and Van Ranst, 2013
*Canis familiaris Papillomavirus 9*	CPV9	*ChiPV*	*ChiPV 1*	Rector and Van Ranst, 2013
*Canis familiaris Papillomavirus 10*	CPV10	*ChiPV*	*ChiPV 3*	Rector and Van Ranst, 2013
*Canis familiaris Papillomavirus 11*	CPV11	*ChiPV*	*ChiPV 1*	Rector and Van Ranst, 2013
*Canis familiaris Papillomavirus 12*	*CPV12*	*ChiPV*	*ChiPV 1*	Rector and Van Ranst, 2013
*Canis familiaris Papillomavirus 13*	CPV13	*TauPV*	*TauPV 2*	Munday et al., 2016; Rector and Van Ranst, 2013
*Canis familiaris Papillomavirus 14*	CPV14	*ChiPV*	*ChiPV 3*	Rector and Van Ranst, 2013
*Canis familiaris Papillomavirus 15*	CPV15	*ChiPV*	*ChiPV 3*	Rector and Van Ranst, 2013
*Canis familiaris Papillomavirus 16*	CPV16	*ChiPV*	*ChiPV* 2	Munday et al., 2016
*Canis familiaris Papillomavirus 17*	CPV17	*TauPV*	*TauPV 1*	Munday et al., 2016
*Canis familiaris Papillomavirus 18*	CPV18	ChiPV	*ChiPV* 1	Lange et al., 2016
*Canis familiaris Papillomavirus 19*	CPV19	*TauPV*	*TauPV 1*	Tisza et al., 2016
*Canis familiaris Papillomavirus 20*	CPV20	*ChiPV*	*ChiPV* 1	NCBI
*Canis familiaris Papillomavirus 21*	CPV21	*TauPV*	-	NCBI
*Canis familiaris Papillomavirus 22*	CPV22	*TauPV*	_	NCBI
*Canis familiaris Papillomavirus 23*	CPV23	*TauPV*	_	NCBI

**Figure 2 -:**
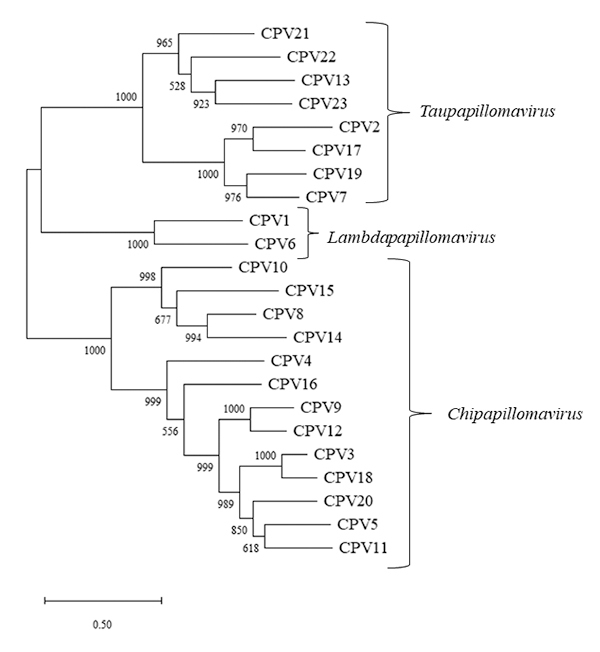
Midpoint maximum likelihood phylogenetic tree based on L1 nucleotide sequences of CPV. Branch support was assessed with 1000 bootstrap replicates. The evolutionary model of nucleotide substitution was the TVM+I+G, selected by jModelTest. Bar scale represents the nucleotide substitution per site. Each type of CPV is grouped together in genus-based clusters: *Taupapillomaviru*s, *Lambdapapillomavirus* or *Chipapillomavirus*.

*PVs* are named according to the scientific name of the host and the type of papillomavirus identified. The canine papillomavirus is therefore named “*Canis familiaris Papillomavirus*” (CPV) with the identification number of the type added to the end of the name, except in the case of CPV1 which is known as “*Canis familiaris* oral Papillomavirus” (Bernard et al., 2010). In the database *Papillomavirus Episteme* (PaVE), the 23 CPV types identified to date are distibuted into three genera: *Lambdapapillomavirus (LambdaPV)* (CPV 1, 6), *Taupapillomavirus (TauPV)* (CPV 2, 7, 13, 17, 19, 21, 22, 23) and *Chipapillomavirus (ChiPV)* (CPV 3-5, 8-12, 14-16, 18, 20) (Figure 2).

## Genetic diversity and pathogenicity

PV types, subtypes and variants may differ in virulence and influence host disease development (Lange and Favrot, 2011). In dogs, for example, lesions caused by CPV can differ clinically according to the type of *PV* with which they were infected (Zhou et al., 2014). For comparison purposes, there are more than 220 identified types of HPV in the *PaVE* database, while, so far, only 23 types of CPV have been identified (Bernard et al., 2010). There is, therefore, a need for more studies focusing on the analysis of CPV genetic diversity.

CPV have been identified worldwide (Sykes and Luff, 2014); Table 3 shows the different CPV types with their associated lesion and the country of origin. HPV studies show that different etiologies, risk factors, and the prevalence of a viral type may be associated with its geographic origin (Sabattini et al., 2016). Information on the geographical distribution of CPVs is still scarce, but more data in this area could contribute to a better understanding of the risk factors related to infection with particular types. As certain types of CPV have been confirmed as risk factors for malignant skin lesions, the risk of cancer development may vary according to the prevalence and geographical distribution of these CPV types.

**Table 3 -:** Geographical distribution of CPV types and their associated lesions in dogs per country of identification.

Country	CPV type	Lesions	GenBank ID	References
Mexico	CPV	CTVT	_______	Ayala-Díaz et al., 2019
Brazil	CPV1	Exophytic oral and cutaneous papillomas	KF199909	Alcântara et al., 2014
Brazil	CPV1	Exophytic oral and cutaneous papillomas; oral SCC in situ	MF321769 - MF321777	Reis et al., 2019
Italy	CPV1	Oral SCC	_____	Porcellato et al., 2014
Italy	CPV1	Oral, cutaneous, tonsillar - SCC	_____	Sabattini et al., 2016
Italy	CPV1	Endophytic papillomas	GQ204117	Lange et al., 2010
Korea	CPV1	Oral papillomas	_____	Yhee et al., 2010
Turkey	CPV1	Oral papillomatosis	_____	Sancak et al., 2015
Turkey	CPV1	Oral e cutaneos papillomatosis	KY445587 - KY445599	Oğuzoğlu et al., 2017
Germany	CPV1	Oral papillomas, oral SCC	_____	Teifke et al., 1998
South Korea	CPV1	Oral cancer	FJ479789.1	Unpublished
South Africa	CPV1	Oral papilloma	KX587461.1	Regnard et al., 2016
Switzerland	CPV1	Assymptomatic	_____	Lange et al., 2011
China	CPV1	Oral papilloma	HM054515.1	Unpublished
Taiwan	CPV1	Oral papilloma	MN617831-33	Chang et al., 2020a
Taiwan	CPV1	Digital papilloma	MN617834	Chang et al., 2020a
USA	CPV1	SCC	_____	Thaiwong et al., 2018
Taiwan	CPV2	Papilloma inthe elbow	MN606026	Chang et al., 2020a
Germany	CPV2	Endophytic papillomas	GQ204118	Lange et al., 2010
USA	CPV2	Footpad lesions, endophytic papilloma	NC_006564	Yuan et al., 2007
Japan	CPV2	Papilloma on footpads	LC363559	Iyori et al., 2019
USA	CPV3	Pigmented plaques	_____	Luff et al., 2012b
Switzerland	CPV3	Epidermodysplasia verruciformis, in situ SCC	NC_008297	Tobler et al., 2006
USA	CPV3	Pigmented plaques, SCC	______	Thaiwong et al., 2018
USA	CPV4	Pigmented plaques	_____	Luff et al., 2012a
Switzerland	CPV4	Pigmented plaques	NC_010226	Unpublished
Japan	CPV4	Pigmented plaques	LC489227-29	Yu et al., 2019
Australia	CPV4	Saliva samples	MK205376-79	Bhatta et al., 2019
Germany	CPV5	Pigmented plaques	FJ492743	Lange et al., 2009
USA	CPV5	Pigmented plaques	_____	Luff et al., 2012b
Switzerland	CPV6	Endophytic papillomas	GQ204119	Lange et al., 2010
Taiwan	CPV6	Digital inverted papilloma	MN606027	Chang et al., 2020a
Taiwan	CPV6	Papilloma in the paw	MN606028	Chang et al., 2020a
Switzerland	CPV6	Endophytic papillomas	FJ492744	Lange et al., 2009
Scotland	CPV7	Exophytic papillomas, SCC	FJ492742	Lange et al., 2009
Switzerland	CPV8	Pigmented plaques	HQ262536	Lange et al., 2012b
Australia	CPV8	Saliva sample	MK205381	Bhatta et al., 2019
New Zealand	CPV9	Pigmented plaques	GU220384	______
USA	CPV9	Pigmented plaques	JQ040505	Luff et al., 2012b
USA	CPV9	Pigmented plaques	JF800656	Yuan et al., 2012
Taiwan	CPV9	Digital papilloma; inguinal SCC	MN606029	Chang et al., 2020a
Taiwan	CPV9	Cutaneous papilloma	MN606030	Chang et al., 2020a
Japan	CPV9	Skin pigmented plaque	MT265226	Chang et al., 2020a
Taiwan	CPV9	SCC	MT265225	Chang et al., 2020a
Switzerland	CPV9	Pigmented plaques	JQ701801	Lange et al., 2013
USA	CPV10	Pigmented plaques	JQ040504	Luff et al., 2012b
USA	CPV10	Pigmented plaques	NC_016075	Luff et al., 2012a
USA	CPV11	Pigmented plaques	JF800658	Zhou et al., 2014
USA	CPV11	Pigmented plaques	JQ040501	Luff et al., 2012b
USA	CPV12	Pigmented plaques, SCC	_____	Luff et al., 2016
USA	CPV12	One pigmented plaque	JQ754321	Zhou et al., 2015
USA	CPV12	Pigmented plaques	JQ040502	Luff et al., 2012b
USA	CPV12	Footpad lesions	KX817182	Anis et al., 2016
Switzerland	CPV13	Oral papillomatosis	JX141478	Lange et al., 2012a
Switzerland	CPV14	Pigmented plaques	NC_019852	Lange et al., 2013
USA	CPV15	Pigmented plaques	JQ040503	Luff et al., 2012b
Taiwan	CPV15	Digital verrucous scc	MN606031	Chang et al., 2020a
Taiwan	CPV16	Inguinal SCC	MN606032	Chang et al., 2020a
Taiwan	CPV16	Dysplasia of squamous ephiyelium	MN606033	Chang et al., 2020a
Taiwan	CPV16	SCC	MN606034; MN606035	Chang et al., 2020a
USA	CPV16	Pigmented plaques, SCC	KP099966	Luff et al., 2016
Brazil	CPV16	Pigmented plaques; in situ and invasive SCC	MG009510	Alves et al., 2020
USA	CPV16	Pigmented plaques, SCC	___	Thaiwong et al., 2018
New Zealand	CPV17	Oral SCC	KT272399	Munday et al., 2016
Australia	CPV17	Saliva samples	MK205383-91	Bhatta et al., 2019
USA	CPV18	Pigmented plaques	JQ040499	Luff et al., 2012b
USA	CPV18	Pigmented plaques	KT326919	Lange et al., 2016
Japan	CPV18	Pigmented plaques	LC489230-31	Yu et al., 2019
USA	CPV19	Oral papilomatosis	KX599536	Tisza et al., 2016
USA	CPV20	_	KT901797	Unpublished
USA	CPV21	Respiratory infection signs	MH285952	Altan et al., 2019
USA	CPV22	Respiratory infection signs	MH285953	Altan et al., 2019
USA	CPV23	Respiratory infection signs	MH285954	Altan et al., 2019

The first *Canis familiaris* oral Papillomavirus 1 (CPV1) was identified in 1994, and remains the most commonly detected type worldwide (Delius et al., 1994; Sancak et al., 2015). Since then, several sequences of CPV1 have been deposited in public databases, which has shown the genome to be highly conserved, which may influence the nature of infection (Regnard et al., 2016). CPV1 is known to cause oral papillomas, but has also been detected in ocular conjunctiva epithelial hyperplastic lesions, cutaneous papillomas and is present in asymptomatic dogs (Brandes *et al*., 2009; Lange and Favrot, 2011; Lange *et al*., 2011; Sancak *et al*., 2015). 

Furthermore, other types have been identified in the oral cavity, namely CPV types 2, 4, 8, 13, 17 and 19 (Munday et al., 2016; Tisza et al., 2016; Lange et al., 2019). In the USA, CPV2 and CPV19 were identified together with CPV1 in a case of coinfection of oral papillomatosis (Tisza *et al.*, 2016). Another study demonstrated the coinfection of canine papillomavirus with CPV1 and CPV2 together but in different lesions: cutaneous and eyelid conjunctiva papillomas (Lange *et al*., 2019). CPV13 and 17 were identified in samples from New Zealand and Switzerland, respectively. CPV4, CPV8 and CPV17 were detected in samples of dog saliva from Australia (Table 3).

Oral papillomatosis is most common in young dogs, and it is manifested by exophytic warts that have a hard consistency with a cauliflower, nodular or fringed form (Lange and Favrot, 2011). This kind of lesion can multiply and persist in immunosuppressed dogs, resulting in the worsening of clinical symptoms and pharyngeal obstruction and dysphagia (Fernandes et al., 2009). 

The cutaneous lesions due to CPV infection could be exophytic or endophytic papillomas, which can be differentiated by histopathological examination. CPV types 1, 2, 6, 7, 9 and 12 have been identified in cutaneous papillomas. All these CPV types were related to exophytic papillomas. It should be noted that CPV7 has only been found in cutaneous exophytic lesions associated with malign neoplasia, while CPV9 has only been found in exophytic cutaneous papilloma with generalized verrucosis. Moreover, endophytic papillomas have only been only associated with CPV types 1, 2 and 6 (Lange et al., 2010; Lange and Favrot, 2011; Cavana et al., 2015; Anis et al., 2016; Munday et al., 2016). 

CPV2 is characterized by tropism in the footpad region, with the presence of endophytic papillomas, and has been found in dogs from Germany, Japan and the USA. In addition to CPV2, CPV12 was also found in footpad lesions in dogs from the USA, and other CPV types were identified in endophytic papillomas of dogs from different countries: CPV1 in Italy; and CPV6 in Switzerland and Taiwan (Table 3).

Clinically, endophytic lesions have been described as having distinct cutaneous presentations namely classic greyish cup-shaped nodules 1-2 cm in diameter with a central pore, dome-shaped lesions 4mm in diameter, and black papules 2mm in diameter (Lange et al., 2010). 

Histologically, the changes found in exophytic lesions are epidermal hyperplasia, hyperkeratosis, inclusion bodies, keratohyalin granules in the spinous layer, clear cells, koilocytes (Lange and Favrot, 2011), hyperplasia of the epithelium, hyperpigmentation, hyperkeratosis and keratohyaline granules occurring in the pigmented plaques (Luff et al., 2016). The endophytic lesions show epidermal papillary projections extending into the dermis. Parakeratotic cells, keratohyaline granules, koilocytes, inclusion bodies intranuclear basophilic and eosinophilic may also occur in endophytic lesions, and eosinophilic cytoplasmic inclusions have also been observed (Lange *et al.,* 2009). 

*Lambpapillomavirus* and *Taupillomavirus* are involved in endophytic and exophytic lesions. However, the most recently identified types of *Taupapillomavirus*, CPV 21, 22, 23, were detected in samples from dogs with signs of respiratory infection from metagenomic analysis of the nasal virome (Altan et al., 2019).

Pigmented plaques, another form of disease caused by CPV, are hyperkeratotic, hyperpigmented plaques of up to 3 cm in diameter usually located in the leg and abdomen. All CPV types isolated in benign pigmented viral plaques belongs to the genus *ChiPVs* (Munday and Kiupel, 2010; Lange et al., 2013). 

Pigmented plaques have been described in the USA (CPV 3-5, 9-12, 15, 16, 18), Germany (CPV5), Switzerland (CPV 3, 4, 8, 9, 14), Japan (CPV4, 9, 18), New Zealand (CPV9 and 15), and Brazil (CPV16) (Table 3). CPV18 and CPV4 have been identified in pigmented plaques of Pug dogs, indicating a possible genetic predisposition to the virus (Lange et al., 2016; Yu et al., 2019).

In the pigmented plaques histology, it is possible to observe acanthosis; hyperkeratosis, hyperpigmentation and hyperplasia of the epidermis; clusters of large keratohyaline granules in the spinous stratum; and koilocytes in the stratum granulosum or clear cells (Lange and Favrot, 2011; Lange *et al*., 2013; Yu et al., 2019).

Studies have shown CPV infection to have a self-limiting characteristic. Oral lesions caused by CPV1 can also be self-limiting over a period of one year (Sancak et al., 2015). In addition, a regression of a footpad exophytic lesion due to CPV2 infection has been observed after biopsy (Iyori et al., 2019).

In Mexico, sequences of CPV DNA were identified in 16 of 21 cases of canine transmissible venereal tumor. This tumor is present in the genital organ as a mass and is sexually transmissible. There is no definition of its etiology and further studies are necessary to determine whether CPV is involved in the development of this type of cancer and which type or variant might be responsible for the disease (Ayala-Díaz et al., 2019).

### Cancer - Squamous Cell Carcinoma (SCC)

SCC is a common cancer in dogs, especially oral SCC, which is the second most common neoplasm in the oral cavity of dogs (Munday et al., 2016). However, the etiology of oral SCC in dogs is not yet well established. In humans, for example, about 25% of oral SCC is due to HPV infection (Ryerson et al., 2008). CPV types have been associated with the progression of cutaneous pigmented plaques to SCC (Goldschmidt et al., 2006; Luff et al., 2016). However, some studies have shown that progression is rare and the etiology of canine SCC is still unclear (Porcellato et al., 2014; Munday *et al*., 2015a; Sabattini et al., 2016).

The development of cutaneous SCC, with the presence of PV antigens detected by immunohistochemistry (IHC), occurred in dog at sites where vaccines of live CPV1 was injected (Bregman et al., 1987). The first report of oral SCC caused by CPV1 was demonstrated by IHC in 1998, with the progression of the lesion in the oral cavity (Teifke et al., 1998). The presence of CPV1 DNA in oral SCC has been demonstrated in other studies, but how the virus acts in the progression to cancer has yet to be determined (Porcellato et al., 2014; Sabattini et al., 2016). Some studies have reported malignant transformation and an increase of CPV1 associated with SCC in the last ten years, suggesting that CPV1 could be responsible for this lesion and its progression to cancer (Ibarra et al., 2018; Thaiwong et al., 2018; Chang et al., 2020a).

Progression to metastatic SCC caused by CPV2 was present in the endophytic lesions of dogs with severe combined immunodeficiency, the result of a mutation in the common gamma chain (Goldschmidt et al., 2006). The progression of multiple pigmented skin plaques into metastatic SCCs was demonstrated in dogs infected with CPV12 and CPV16 (Luff et al., 2016). As occurs in high-risk HPV types, the CPV16 genome has been found to be integrated into the host chromosome in a case of metastatic SCC (Luff *et al*., 2019).

CPV17 was found in a case of multiple oral SCCs, with increased expression of p16CDKN2A protein (p16). This protein has been used as a molecular marker to demonstrate the etiology of the HPV infection in SCC development (Munday et al., 2015b; Munday *et al*., 2016). In dogs, the p16 protein has not yet been associated with the etiology of CPV in SCCs, due to the absence of CPV DNA in dogs with this type of cancer and increased expression of p16, and the absence of p16 in some cases of SCC with CPV DNA (Munday *et al*., 2015a; Sabattini et al., 2016). 

Histological examination has shown the malignant transformation of benign lesions due to CPV1, CPV3 and CPV16 infection. Both expression of p53 and p16 was analyzed simultaneously in the same lesions, but both the benign and the malignant lesions had immunoreactivity, making it impossible to identify the same association found in HPV cases, where the immunoreactivity is associated with cancer (Thaiwong et al., 2018). Therefore, further studies are required to identify the mechanisms associated with this progression to cancer. CPV types of all genera have already been identified in cases of SCC: CPV1 (*Lambdapapillomavirus*); CPV2 (*Taupapillomavirus*) and CPV 3, 7, 9, 12, 15, 16, 17 (*Chipapillommavirus*) (Teifke et al., 1998; Goldschmidt et al., 2006; Munday and Kiupel, 2010; Lange and Favrot, 2011; Munday *et al*., 2015b; Luff et al., 2016; Munday *et al*., 2016; Thaiwong *et al*., 2018; Chang et al., 2020a,b). 

Studies on HPV have already demonstrated that some specific HPV types are associated with different risks of cancer development, and that some variant lineages are related to increased risk of cervical cancer development when compared to other HPV lineages in different regions of the world. For example, there are some HPV16 and/or HPV18 lineages and sublineages that are associated with an increased risk of cancer (Xi et al., 2007; Bernard et al., 2010; Van Doorslaer, 2013); The HPV16 European variants (lineage A), for example, are associated with less risk of invasive cancer than the HPV16 lineages B, C and D from other parts of the world. On the other hand, non-European HPV18 variants (lineages B and C) seems to be more related to a higher risk of cervical cancer development than European HPV18 variants (lineage A) (Chen et al., 2011; Cullen et al., 2015). 

To date, this relationship between the lesions and CPV variants (lineages and sublineages) has been little explored. Some studies have identified different isolates or their DNA sequences which differ from the CPV reference type genome; however, they did not discuss whether these isolates could be CPV variants or not, or their potential role in pathogenesis. For example, different isolates of CPV1 were identified in cases of dogs with oral and cutaneous lesions in Turkey and Brazil (Alcântara et al., 2014; Oğuzoğlu et al., 2017; Reis et al., 2019). In Brazil, samples from different regions of the country have shown variants of CPV1 associated with oral and cutaneous lesions (Alcântara *et al*., 2014; Reis *et al*., 2019). In one case of *in situ* oral SCC in a dog from Brazil, a new CPV1 variant was identified, showing the importance of studies that focus on the discovery of CPV variants that may influence the disease and could be associated with cases of cancer (Reis *et al*., 2019). 

Another factor that is involved in the development of cancer is the expression of the E5, E6 and E7 oncoproteins, which is well known in the HPV infection, but is still unclear in CPV infection. A comparative study with benign and malignant epithelial neoplasia SCC due to CPV9 infection in dogs showed that there is no difference in the mRNA expression of E6 and E7 genes between the benign and malignant lesions. In the same study, it was shown that the E2 protein may influence the development of cancer in lesions caused by CPV9 by the deletion of a nucleotide sequence (Chang et al., 2020b). Therefore, further studies must be carried out to investigate the possible role of CPV proteins involved in cancer development.

## Conclusions and future perspectives

Given the fact that HPV in humans is associated with different levels of cancer risk, including a high risk in respect of specific types and variants of HPV, it is reasonable to assume that some types of CPV may also be associated with cancer. A study showing a putative CPV1 variant in a case of oral SCC in situ highlights the importance of the genetic characterization of nucleotide sequences of CPV, identifying the variants that could be more pathogenic and related to cases of cancer around the world.

Studies of CPV genetic diversity are mostly about discovery of new types. However, in order to increase our knowledge in respect of the development of cancer caused by CPV it is important that future studies also focus on the identification and characterization of CPV subtypes and variants, their association with SCC, the expression of genes involved in the progression to cancer, and the epidemiological characteristics of the genetic variants associated with pathogenic aspects. 
